# Case series of nebulizing r‐tPA for COVID‐19 induced acute respiratory distress syndrome

**DOI:** 10.1002/ccr3.6283

**Published:** 2022-09-05

**Authors:** Shahideh Amini, Zohre Labbani‐Motlagh, Rasoul Aliannejad, Seyed Mohammad Pourabbas, Mohammad Vasei

**Affiliations:** ^1^ Department of Clinical Pharmacy, Faculty of Pharmacy Tehran University of Medical Science Tehran Iran; ^2^ Advanced Thoracic Research center Tehran University of Medical Science Tehran Iran; ^3^ Department of Clinical Pharmacy Tehran University of Medical Science Tehran Iran; ^4^ Department of Internal Medicine, Shari'ati Hospital Tehran University of Medical Science Tehran Iran; ^5^ Cell‐Based Therapies Research Center, Digestive Disease Research Institute, Shari'ati Hospital Tehran University of Medical Science Tehran Iran

**Keywords:** ARDS, COVID‐19, fibrinolysis, recombinant tissue plasminogen activator, r‐tPA

## Abstract

Fibrin deposition in the alveolar spaces during pulmonary involvement of COVID‐19 impairs the O_2_/CO_2_ exchange and leads to respiratory symptoms. In this report, Recombinant Tissue Plasminogen Activator (r‐tPA) has been nebulized to 3 critically ill COVID‐19 patients in order to resolve the deposited fibrin while avoiding the risk of bleeding. Based on these observations, nebulization of r‐tPA may be a potential therapeutic approach and new area of research for future studies.

## Graphical abstract

Fibrin cast depositions play an important role in COVID‐19‐induced ARDS. Systemic administration of fibrinolytic may accompany with risk of bleeding. Nebulization of r‐tPA (Recombinant Tissue Plasminogen Activator) can temporally associated with transient improvements in oxygenation in COVID‐19‐induced ARDS. Fibrinolytic nebulization would minimize bleeding risk‐associated systemic administration.

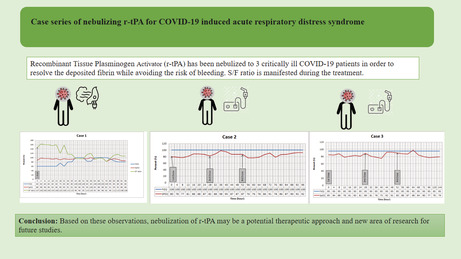

## 1 INTRODUCTION

Infection with the novel coronavirus, SARS‐CoV‐2, can lead to different severity of pulmonary involvements. Nevertheless, mortality of SARS‐CoV‐2 infection arises mainly through the development of viral pneumonia‐induced acute respiratory distress syndrome (ARDS), sepsis, and multi‐organ failure.^1^ Regardless of insulting agent and despite the high rate of mortality, ARDS has no definite treatment. It can just be managed by supportive care and lung‐protective mechanical ventilation, prone positioning.^1^, ^2^, ^3^, ^4^

The exact pathophysiological process associated with COVID‐19 ARDS remains unclear, endothelial dysfunction, cytokine storm, and hypercoagulability state have been defined as the main hyper inflammatory and coagulation dysregulation.^1^ Dysregulation of the coagulation and fibrinolytic systems provoke fibrin deposition which are found in the lungs of COVID‐19‐induced ARDS.^5^, ^6^ Same as SARS (Severe Acute Respiratory Syndrome), along with high C‐reactive protein level and platelet infiltrations in lung, high levels of tissue growth factor‐β in infected cells lead to overproduction of extracellular matrix metalloprotease inhibitors, such as Plasminogen Activator Inhibitor‐1 (PAI‐1). PAI‐1 is the main fibrinolytic inhibitor and independently correlates with mortality.^7^ PAI‐1 decrease endogenous local fibrinolytic activity in airspaces, and thus, places the patient in hyper‐coagulable and hypo‐fibrinolytic state at the same time.^7^ Plasma levels of coagulation markers, such as D‐dimer and fibrinogen, are elevated among the COVID‐19 patients and fibrin deposition is seen in alveolar spaces and lung parenchyma.^8^ Postmortem lung biopsies from patients with COVID‐19‐induced ARDS showed accumulation of monocyte and macrophage in fibrin deposits. Fibrin deposition can be found in an infected patient before pneumonia signs manifest.^7^ According to hypercoagulability tendency, prophylactic or therapeutic use of anticoagulants has been recommended with different guidelines.^7^ Even though, prophylactic or therapeutic use of heparin may prevent new fibrin formation and reduces inflammatory markers among this population, it will not be affective in pre‐existing fibrin depositions in the air spaces and also it is not able to degrade pre‐existing microthrombosis in the lung.^5^, ^9^ Administration of plasminogen activator medications in animal models of trauma and sepsis has been shown to be effective in preventing development of ARDS.^10^ Furthermore, Tissue Plasminogen Activator showed anti‐inflammatory properties by inhibiting neutrophil activation.^7^, ^11^ In addition to limited effective treatment in patients with COVID‐19‐induced ARDS alongside to high mortality, the pharmacotherapy approach based on pathophysiological findings might be helpful. Accordingly, in our assumption, recombinant Tissue Plasminogen Activator (r‐tPA) administration could affect both inflammatory and coagulation dysregulation of ARDS associated with COVID‐19.

In this study, we assessed the pulmonary physiologic markers of three patients with COVID‐19 after nebulizing r‐tPA individually.

## 2 METHODS AND PROTOCOL OF r‐tPA NEBULIZING

This assessment was approved by ethics committee of Tehran University of Medical Science (IR.TUMS.VCR.REC.1399.025) and was performed in Shariati Hospital, Tehran, Iran. Patients with diagnosis of COVID‐19 based on Chest computed tomography (CT) and positive polymerase chain reaction (PCR) and PaO_2_/FiO_2_ (PF ratio) less than 100 enrolled for this assessment.

They received all of common treatments based on our institutional protocol. Jet nebulizer in all (including 7 ml capacity container, mouth piece, and T‐piece) was supplied from Saramad Teb Parayeh Company and r‐tPA Actilyse® treatment set, Batch number 003202, were used during this study. Fifty mg of Actilyse® dissolved in 50 ml of the solvent (1 mg/ml solution) and nebulized by jet nebulizer in divided volumes over 2 h. Oxygen saturation (SpO_2_) and fraction of oxygen inspired (FiO_2_) recorded every 4 h. Next dose administered at 24‐h interval.

## 3 CASE REPORT

### 3.1 Case 1

Forty‐seven‐year‐old woman presented to emergency department (ED) with complaint of dry cough, asthenia, fever, and myalgia for 7 days. Her past medical history was positive for hypertension, and she was taking losartan 25 mg twice daily. At presentation, general physical examination revealed a blood pressure (BP) 110/80 mmHg, respiratory rate (RR) 24 resp/min, pulse rate (PR) 105 bpm, SpO_2_ 80% at room air and oral temperature of 41°C. In auscultation, rales were heard in lower lobes of lung and physical exam of abdomen and extremities were normal. Chest CT was taken and bilateral multifocal dominantly peripheral patchy ground glass opacities and consolidations which were highly suggestive of COVID‐19 pneumonia were seen. Laboratory data reported lymphocyte count 1196 cells/mm^3^, hemoglobin 6.6 g/dl, mean corpuscular volume (MCV) 61 fl, CRP 80 mg/L, erythrocyte sedimentation rate (ESR) 31 mm/h, and Lactic acid dehydrogenase (LDH) 725 U/L abnormal.

**FIGURE 1:**
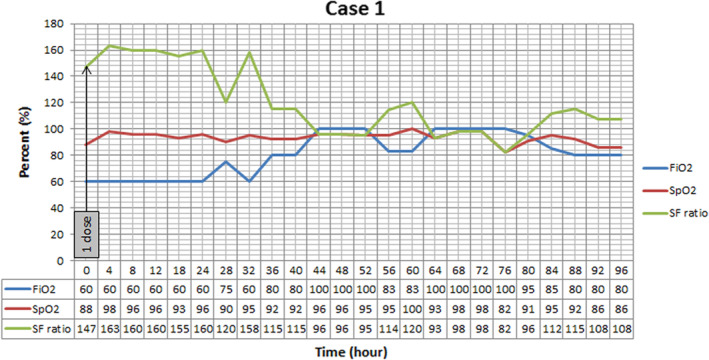
Oxygenation trend in Case 1 after nebulizing one dose of recombinant Tissue Plasminogen Activator (r‐tPA). As it is shown, SpO_2_ start to increase 4 h after starting the treatment. Positive end expiratory pressure was set at 5 cmH2O, and patient was placed at supine position.

Patient admitted to ward and oxygen supplementation with reservoir bag mask, heparin 5000 units SC every 12 h, hydroxychloroquine 400 mg PO stat then 200 mg twice daily and lopinavir/ritonavir 200/50 mg 2 tablets PO twice daily, cefepime 1 g IV three times a day, levofloxacin 750 mg IV daily, and vancomycin 1.5 g IV stat and 1 g twice daily were ordered. Oxygen saturation progressively declined and patient was not able to tolerate continuous positive airway pressure (CPAP). Fever continued and imipenem 1 g three times a day substituted for cefepime. Hemoglobin begins to fall without any distinct source of bleeding. After 2 days, her oxygen saturation falls to 60% on 15 L reservoir bag mask, she became agitated and medical team decided to intubate her. Due to low SpO_2_, she received 250 mg methyl prednisolone after intubation. The nebulizing r‐tPA was ordered on the sixth day after admission. Before r‐tPA administration, SpO_2_ 88% on FiO_2_ 60% was recorded. One dose was nebulized based on study protocol and oxygenation trend (SpO_2_, FiO_2_) was recorded every 4 h (Figure 1). At the end of the day, oxygenation improved and SpO_2_ reached 96% despite constant FiO_2_ of 60%.

Unfortunately, at the time of the next dose, manifestation of seizure and upward gaze was detected in the patient; therefore, treatment was stopped, imipenem discontinued and antiepileptic agents started. Considering the clinical situation of the patient brain CT was not feasible. From 1 day after nebulizing, oxygen saturation began to decline. Seizure and seizure‐like events was not noticed again. Finally, 9 days later, patient expired due to asystole after 45 min of CPR.

### 3.2 Case 2

A 78‐year‐old man with past medical history of diabetes mellitus (on oral medication) and hypertension admitted to ED, complaining dry cough, fever, and dyspnea for 1 week. His vital signs at presentation were SpO_2_ 78%, RR 25 resp/min, PR 105 bpm, BP 160/90 mmHg, and oral temperature of 37.5°C. Chest CT findings were compatible with COVID‐19 pneumonia. Out of range laboratory data noticed for lymphocyte count 693 cells/mm3, CRP 87 mg/L and ESR 82 mm/h.

**FIGURE 2:**
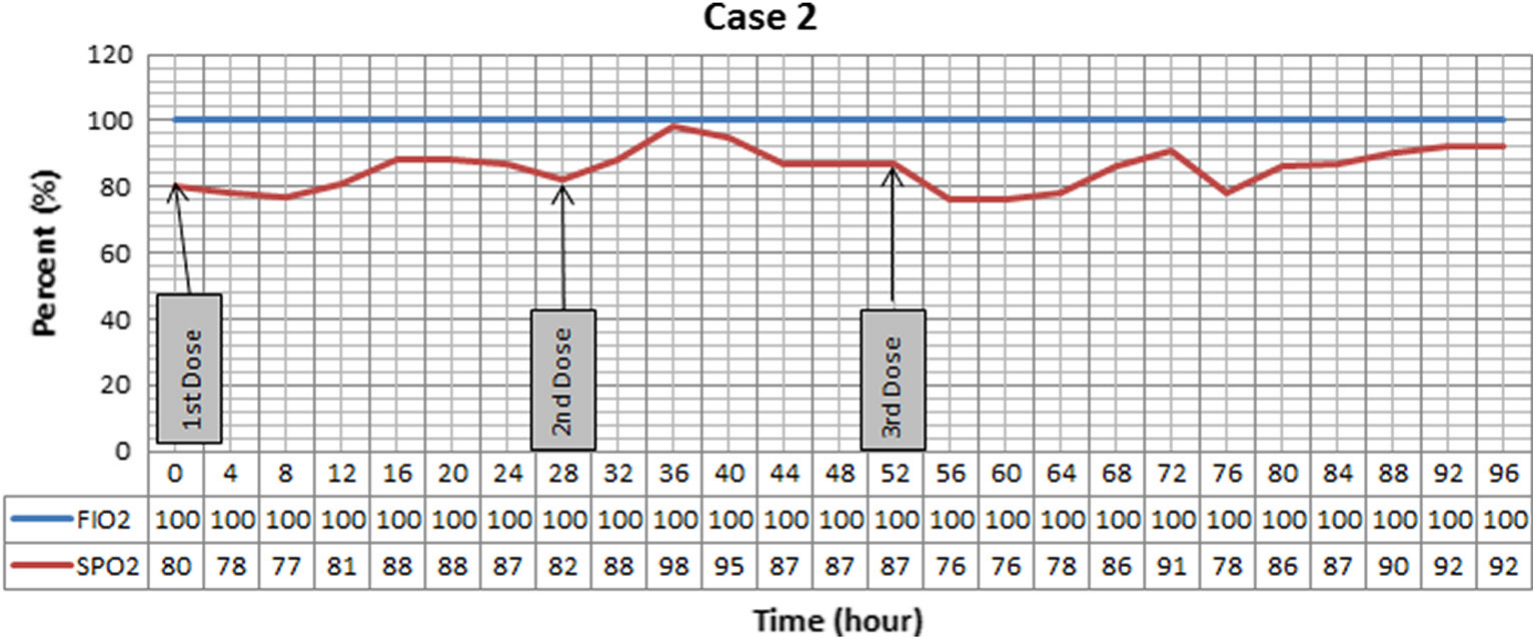
Oxygenation trend in Case 2 after nebulizing three doses of recombinant Tissue Plasminogen Activator (r‐tPA). Saturation responds to treatment and reaches to 98% during the course of study. Positive end expiratory pressure was set at 5 cmH2O, and patient was placed at supine position.

Pressure support ventilation, scheduled prophylactic doses of heparin, methyl prednisolone 125 mg daily for 3 days, hydroxychloroquine 400 mg PO stat then 200 mg twice daily, and lopinavir/ritonavir 200/50 mg 2 tablets twice daily started for him. However, oxygenation progressively exacerbated, and 14 days after admission due to severe respiratory distress (SpO_2_ 60% on reservoir bag mask, PR 160 bpm), intubation was inevitable. At day 18th of admission, r‐tPA nebulization was ordered because of low oxygen saturation (80%) despite 100% FiO_2_. Three doses were administered in three consecutive days according to our study protocol. As it evident in the Figure 2, oxygenation became better after every nebulization and reached 91% saturation after 3 days. Afterward oxygenation exacerbated with tendency to fall and ventilator associated pneumonia with resistant microorganisms developed. SF ratio is not presented in Figure 2 due to constant FiO_2_ and similar pattern of changes with SpO_2_. Even though all the efforts were made by medical team, patient expired on 38th day of admission after 45 min of CPR.

### 3.3 Case 3

A 65‐year‐old woman referred to ED, complaining exertional dyspnea and asthenia. Medical records showed history of diabetes mellitus on oral agent and anemia. Vital signs at presentation were SpO_2_ 85% at rest on room air, PR 90 bpm, BP 148/72 mmHg, RR 30 resp/min, and temperature 36.5°C. Imaging and PCR were positive for COVID‐19 pneumonia. Except CRP 84, out of range laboratory data noticed for lymphocyte count 693 cells/mm^3^, CRP 87 mg/L, and ESR 82 mm/h. ESR 107 mm/h and LDH 1249 U/L other laboratory findings were in normal range.

**FIGURE 3:**
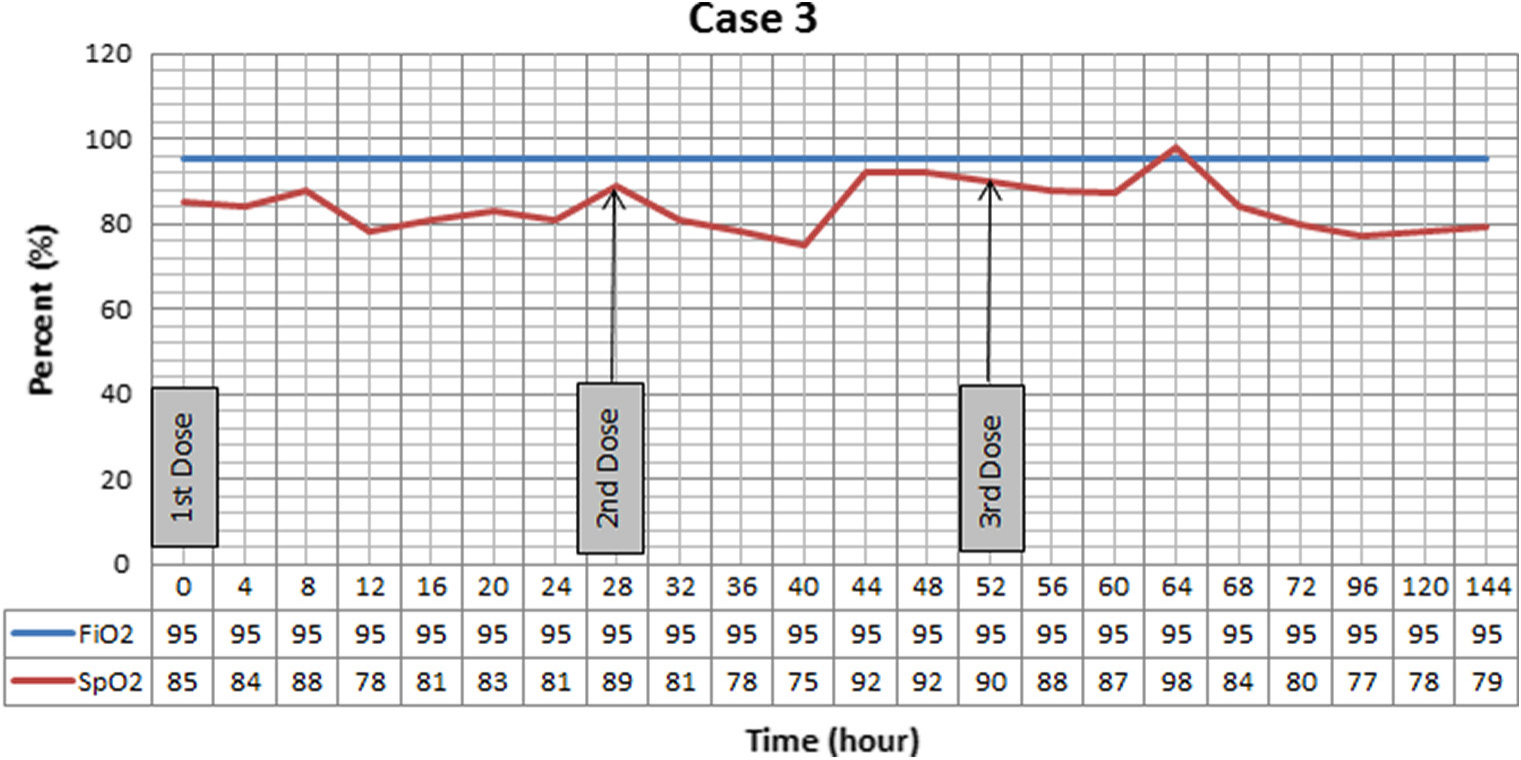
Oxygenation trend in Case 3. While administering recombinant Tissue Plasminogen Activator (r‐tPA) oxygenation improved. After stopping the nebulization oxygen saturation felt to lower than baseline. Positive end expiratory pressure was set at 5 cmH2O, and patient was placed at supine position.

Patient admitted to COVID‐19 ward and institution treatment protocol was performed for him (Hydroxychloroquine 400 mg PO stat then 200 mg twice daily, sofosbuvir/daclatasvir 400/60 mg daily, heparin 5000 unit SC twice daily and CPAP for 3 h three times a day). He did not tolerate CPAP and oxygen saturations continued to decline. Methyl prednisolone 125 mg plus 100 mg thiamin and 6 g of vitamin C ordered for 3 days. On 13th day of admission r‐tPA nebulization started. Oxygenation trend presented on Figure 3, baseline SpO_2_ was 85% on reservoir bag mask, it improved during period of study and reached to maximum 98% after 3 days. Next day by stopping r‐tPA nebulization, oxygen saturation felt to 77%. SF ratio is not presented in Figure 2 due to constant FiO_2_ and similar pattern of changes with SpO_2_. Twenty‐three days after admission, patient became intubated because of respiratory arrest, and at 25th day, expired due to asystole after 45 min of CPR.

Demographic data and the impact of treatment are summarized in the Table 1.

**TABLE 1:** Summary of patients' characteristics

	Case 1	Case 2	Case 3
Age (years)	47	78	65
Gender	Female	Male	Female
Weight (kg)	70	70	70
Height (cm)	158	160	158
PMH	Hypertension	Diabetes mellitus Hypertension	Diabetes mellitus Anemia
Duration of symptom before admitting (days)	7	7	3
Duration of symptom before r‐TPA (days)	16	25	19
Duration of high flow oxygenation before r‐TPA (days)	6	14	13
Duration of mechanical ventilation before r‐TPA (days)	4	4	0
SF ratio baseline	146	80	89
Compliance baseline	31	19	N/A^a^
Number of doses	1	3	3
SF ratio after treatment	160	91	103

*Note:* Summary of cases. SF ratio: SpO_2_/FiO_2_.

Abbreviations: PMH, Past Medical History; r‐TPA, recombinant Tissue Plasminogen Activator.

^a^ Not applicable.

## 4 DISCUSSION/CONCLUSIONS

In all three cases, we have seen improvement in SF ratio as an oxygenation index. However, the effect of treatment was short lasting and after stopping r‐tPA nebulization these effects vanished. In the first case, nebulization led to +9% changes at the end of the day. The second case, showed +10%, +22%, and 13% improvement compared with baseline after each dose, respectively. Continuous improvement was seen in the third case, as +4.7%, +8.2%, and +15.2% variation occurred after every nebulization. However, by discontinuing the treatment, fall in SF ratio in all cases was detected.

Fibrin deposition in the alveolar spaces and pulmonary parenchyma and micro‐thrombi formation in the lung vessels is the main histopathologic findings in post‐mortem studies of patients with ARDS. These findings have been observed among patients with COVID‐19‐induced ARDS. Besides coagulopathy manifestation of ARDS, COVID‐19 could lead to endothelial disruption, microangiopathy with thrombosis of capillary vessels and angiogenesis.^12^ Higher levels of schistocytes are introduced as a marker of endothelial injury, erythrocyte exposure to fibrin strands, high probability of micro‐thrombi formation and incidence of multi‐organ failure in COVID‐19 patients.^13^ This probably aggravates micro‐thrombi formation in pulmonary arterioles in patients with ARDS. Although anticoagulation leads to lower levels of inflammatory markers and prevention of thrombosis, it could not disintegrate fibrin casts in ARDS patients broncho‐alveolar lavage.^14^

Based on animal models of ARDS, TPA administration leads to dissolve fibrin deposition and reduction of inflammation by preventing neutrophil activation^15^ Administration of r‐tPA in ARDS patients was evaluated in previous studies. Gram et al. combined intravenous (20 mg) and nebulized (30 mg) of r‐tPA over 2 h. Additionally, nebulization of Heparin (15,000 IU/day) was continued for 13 days. The pulmonary gas exchange of the patient improved significantly.^9^ Intravenous administration of r‐tPA 25 mg over 2 h and 25 mg over 22 h made pulmonary status stable in three COVID‐19‐associated ARDS patients. Based on the findings of this study, even intravenous r‐tPA administration cause short lasting effect and just one patient experienced durable status.^8^ Price et al. have administered 50 to 90 mg intravenous r‐tPA in patients with concomitant COVID‐19‐associated ARDS and pulmonary thromboembolic disease at median 16 days of initiation of symptoms. This study observed better PF ratio following r‐tPA administration.^8^, ^16^ Based on these small reports, Phase IIa STARS trial evaluated the efficacy and safety of intravenous use of r‐tPA in a greater population of SARS‐CoV‐2‐induced ARDS. This study showed improvement in oxygenation in patients who received bolus r‐tPA plus full anticoagulation with heparin. Although this treatment was not managed to affect the mortality. Also, r‐tPA was only effective in Bolus administration and was not in drip route. However, safety of systemic administration of r‐tPA concomitant with full anticoagulation is evident based on the results of this study and zero case of bleeding during the treatment.^17^

In populations other than ARDS such as plastic bronchitis which fibrin depositions have remarkable role in disease pathophysiology, nebulization of r‐tPA has been evaluated. Instillation of 3.5 mg r‐tPA on fibrin casts during bronchoscopy degraded fibrin deposition in one patient^18^ and nebulization of initial 12 mg then a 10 mg dose 1 h later and finally four 5 mg doses at 2‐h intervals in another patient, leads to successful treatment of plastic bronchitis.^19^

Data regarding efficacy and safety of different routes of administration is sparing. Intrathecal and intravenous routes are more effective than nebulization in animal models of acute lung injury; however, bleeding risk is much lower with the latter and there are no reports of bleeding tendency by nebulization.^1^ Local thrombolytic delivery would increase the fibrinolytic potentials of plasma and dissolve the micro‐thrombi in the distinct location. This also would not pose the patient to systemic bleeding.^11^, ^20^ Nevertheless, no adverse reaction such as bleeding is reported following intravenous and nebulization use of r‐tPA in Wang et al. and Price et al. studies.

Based on our observation, in Case 1, hemoglobin level reduction had happened prior to r‐tPA administration and after convulsions, she was suspicious to intracranial hemorrhage, but it was not confirmed by imaging. Further, she received imipenem which is known to be neurotoxic and epileptogenic; this could be another cause of seizure‐like episode.^21^ COVID‐19 could be another cause of neurologic symptoms and strokes.^22^ Although the causality assessment of seizure in this patient was complex, the likelihood of r‐tPA causing a seizure due to ICH was unlikely albeit it was possible. So, Naranjo Score 3 and possible causality considered for this adverse event. No other reaction had happened in two other cases. To the best of our knowledge, there is no published randomized clinical trial on r‐tPA nebulization in ARDS patients, so convincing evidence on safety of r‐tPA nebulization is lacking. We are eagerly waiting for the results of PACA trial (NCT04356833) on efficacy of this method of care.

PLATyPuS trial (NCT02315898) is the only randomized clinical trial of r‐tPA nebulization on pediatric plastic bronchitis and we should wait for the results of this trial about the safety of this therapeutic modality.

Despite previous evidence to address the benefit of r‐tPA in patients with ARDS, some challenges should be considered including the optimal time, route, duration, and interval of administration. Whether the early treatment or more doses can lead to an increased and constant response is unclear. However, it seems that the nebulizing of r‐tPA is an attractive delivery of fibrinolytic medications. Well‐designed clinical trials of thrombolytic therapy would further perform to show the efficacy and utility of this therapy.

Jet nebulizer is less affected than ultrasonic nebulizer by the physiochemical characteristics of the nebulizing solution. Particle size and flow with jet nebulizer is more suitable for drug delivery to alveoli. Also, less physical and thermal stress is imposed to the solution, so the risk of inactivation or degradation of the medical ingredient would greatly decrease. This is particularly important for protein structures like fibrinolytics.^23^, ^24^ We used jet nebulizer in order to maintain the fibrinolytic activity and maximize the drug delivery to alveolar spaces. However, the amount of r‐tPA delivered to alveoli and optimum rheology of the r‐tPA solution is not yet assessed.

Although interpretation of achieved data based on three cases without control arm is not practicable, we observed transient improvement in oxygenation after r‐tPA nebulization in this study. This benefit was short lasting and depended on treatment continuation. Considering the few number of patients, no placebo arm and lack of physiochemical study on r‐tPA nebulization solution, further well‐designed clinical trials are needed.

## AUTHOR CONTRIBUTIONS

Main idea for this treatment modality proposed by Mohammad Vasei. Rasoul Aliannejad was the physician in‐charge for treatment of these patients. Mohammad Poorabbas was responsible for clinical monitoring and data gathering. Shahideh Amini analyzed and interpreted the achieved data and Zohre Labbani‐Motlagh was a major contributor in writing the manuscript. All authors read and approved the final manuscript.

## FUNDING INFORMATION

This work was supported by The Tehran University of Medical Sciences, Grant No: 47125‐101‐199.

## CONFLICT OF INTEREST

The authors have no conflicts of interest to declare.

## ETHICAL APPROVAL

This assessment was approved by ethics committee of Tehran University of Medical Science (IR.TUMS.VCR.REC.1399.025).

## CONSENT

Written consent form was taken from patients or their first‐degree relatives before enrollment.

## Data Availability

The datasets used during the current study are available from the corresponding author on reasonable request.
